# Introduction of protocols for mass production of *Toxoplasma gondii* tachyzoites of the genotype II PRU strain

**DOI:** 10.1002/ame2.12174

**Published:** 2021-06-04

**Authors:** Mohammad Saleh Bahreini, Mohammad Nohtani, Amir Masoud Salemi, Mehdi Mirzaeipour, Naghmeh Dastan, Sara Bajelan, Qasem Asgari

**Affiliations:** ^1^ Department of Medical Parasitology and Mycology School of Medicine Shiraz University of Medical Sciences Shiraz Iran

**Keywords:** BALB/c, genotype II, PRU strain, tachyzoites, *Toxoplasma gondii*

## Abstract

**Background:**

Few investigations of genotype II of *Toxoplasma gondii,* the most prevalent form of the *Toxoplasma* parasite in humans, have been carried out on due to the rapid conversion of tachyzoites to bradyzoites in its life cycle. The current study aimed to create animal and in vitro models for production of the tachyzoites of the Prugniaud (PRU) genotype II strain.

**Methods:**

To develop an immunocompromised model and obtain tachyzoites of the PRU strain, BALB/c mice were orally treated with dexamethasone (10 mg/kg), cyclophosphamide (36 mg/kg), and cyclosporine (18 mg/kg) from 5 days prior to inoculation. Then, 10‐15 tissue cysts of PRU strain were inoculated intraperitoneally into the mice. The tachyzoites obtained from mice were then cultivated in a HeLa cell culture. The resulting yield of tachyzoites was cryopreserved in 92% fetal calf serum, 8% dimethyl sulfoxide. The infectivity of these tachyzoites was evaluated using in vivo and in vitro examinations.

**Results:**

Numerous tachyzoites were observed in the peritoneal fluid of the immunosuppressed mice within 10‐15 days after inoculation, and many tachyzoites were harvested from the HeLa cell culture. Trypan Blue staining showed 80% viability of the tachyzoites recovered from cryopreservation and this was confirmed by HeLa cell culture. In addition, mice infected intraperitoneally with the recovered tachyzoites presented with cysts in the brain after 2 months.

**Conclusion:**

We have developed an animal model for mass production of *T. gondii* tachyzoites of the PRU strain. This method can provide fresh viable tachyzoites of *Toxoplasma gondii* for use as and when required in future investigations.

## 1 INTRODUCTION

*Toxoplasma gondii* is an obligate intracellular protozoan parasite that can cause severe manifestations of the congenital form of the disease in warm‐blooded animals. It can also play an important role in the mortality of immunocompromised individuals, including HIV‐positive patients.^1^ Sporozoites, tachyzoites, and bradyzoites are invasive forms in *Toxoplasma* infection transmission. The main infection source in cats, a definitive host, is the consumption of contaminated meat. In human beings, toxoplasmosis is mainly caused by ingestion of oocysts present in the feces of infected cats.^2^ In immunocompetent individuals, *T. gondii* usually causes asymptomatic infection, but bradyzoites reside latently in tissue cysts of the brain and skeleton muscles.^3^ When immunity decreases, latent tissue cysts continuously rupture, and parasite conversion is a major cause of mortality in patients with AIDS.^4^, ^5^ Owing to the great importance of the disease, numerous studies, using different strategies, have been undertaken on various aspect of *Toxoplasma* and toxoplasmosis. Tachyzoites and tissue cysts have been the major forms of the parasite used in experimental studies, for example, vaccine production, drug susceptibility, and diagnostic testing set up.^6^, ^7^, ^8^, ^9^, ^10^

Animal models and cell cultures are the usual methods of providing a large number of *T. gondii* tachyzoites.^11^ The major genotypes of *Toxoplasma gondii* are classified based on their virulence in mice.^12^ The genotype I strain is highly virulent in mice and does not readily differentiate into bradyzoites, while genotype II strains immediately develop into the latent phase of the disease and all tachyzoites convert to bradyzoites. Genotype II is predominant in humans, but it is difficult to conduct studies on the genotype II tachyzoites because it cannot be obtained in mice.^13^ The lack of a suitable animal model and in vitro system for obtaining fresh genotype II tachyzoites has led to low use of this strain, even though it is the most common form in humans.^14^ Therefore, the present study aimed to introduce methods for production and maintenance of *Toxoplasma gondii* tachyzoites of the Prugniaud (PRU) genotype II strain using in vivo and in vitro culture systems to provide fresh viable tachyzoites when required.

## 2 METHODS

### 2.1 Parasites and cell culture

*T. gondii* (PRU strain), a gift from Professor Ahmad Daryani (Toxoplasmosis Research Center, Mazandaran University of Medical Sciences, Sari, Iran), was passaged in BALB/c mice. Ten female BALB/c mice (5‐6 months old, weighing 25‐30 g) were infected with 5‐10 *T*. *gondii* cysts (PRU strain) intraperitoneally and then maintained in the Comparative Medical Center of Shiraz University of Medical Sciences for 2 months. The chronic form of the disease developed in the mice and cysts were found in their brain smears after 2 months. After killing the mice using a carbon dioxide jar, their brains were examined for the presence of *Toxoplasm*a cysts. The brains were then homogenized and the number of cysts in 20 µL of the homogenized brains was counted using a light microscope. Based on the count, a suspension containing 10‐15 cysts/mL was prepared in sterile phosphate‐buffered saline (PBS; 0.01 mol/L, pH 7.2), for later inoculation. In this study, the mice were provided with standard dried rodent food and water ad libitum and were housed in temperature‐controlled accommodation (22 ± 2°C, 40‐60% humidity).

The HeLa cell line was provided by the Department of Immunology, Shiraz University of Medical Sciences. The cells were initially grown in RPMI 1640 medium (Sigma, USA) supplemented with 15% Fetal Calf Serum (FCS; Sigma, USA) and 1% Pen Strep (100 units of penicillin and 100 µg of streptomycin, Sigma, USA) (growth medium) at 37°C in a 5% CO_2_ atmosphere. When a confluent monolayer was obtained (60‐80% confluency), the subculture procedures were undertaken using RPMI 1640 medium with 10% FCS and 1% Pen Strep (maintenance medium).^15^

### 2.2 Tachyzoite production following immunosuppression of the mice

Fifteen female BALB/c mice (5‐6 months old, weighing 25‐30g) were orally treated with dexamethasone (10 mg/kg), cyclophosphamide (36 mg/kg), and cyclosporine (18 mg/kg) for 15 days to suppress their immune systems. After 5 days, a suspension containing 10‐15 tissue cysts of PRU strain, prepared as described in the previous section, were inoculated intraperitoneally into the immunosuppressed BALB/c mice. Over consecutive days after inoculation, the mice's peritoneal exudates were stained using Giemsa staining and examined by light microscopy. Once the tachyzoites were seen in the peritoneal fluid, the peritoneal cavity of the infected mice was washed twice using 5 mL of sterile phosphate‐buffered saline (PBS; 0.01 mol/L, pH 7.2) and the cellular debris was removed by centrifuging at 1200 *g* for 10 minutes at 4℃.

### 2.3 Mass cultivation

Flasks (75 cm^2^) were seeded with 9 × 10^5^ HeLa cells in a growth medium. When 70% coverage with a HeLa cell monolayer was achieved, the cultures were infected with tachyzoites from immunosuppressed mice (tachyzoite:cell ratios were 1:10),^16^ and incubated at 37°C with 5% CO_2_ for 6‐8 hours, during which time the active tachyzoites could penetrate the cells. The culture media containing cell debris and inactive tachyzoites were then removed and replaced with fresh culture medium. The cultures were examined by phase contrast microscopy using an inverted microscope to detect ruptured cells as well as the presence of tachyzoites. The cultures were harvested when maximally infected (>5 plaques/field at 400× magnification) and were estimated to have ≥1 × 10^5^ tachyzoites/mL. The tachyzoites and cells in the decanted supernatant were filtered out and washed twice with the maintenance medium at 1200 *g* for 10 min.

### 2.4 Comparison of PRU strain tachyzoites with RH strain

The RH strain of *T. gondii* was obtained from Tehran University of Medical Sciences, Tehran, Iran. The tachyzoites of the RH strain were intraperitoneally injected into five female BALB/c mice. After 5‐6 days, tachyzoites were collected by repeated flushing of the peritoneal cavity using phosphate buffered saline (PBS) at pH 7.2. Then the sizes of 100 tachyzoites of the RH strain and 100 tachyzoites of the PRU strain, obtained as described in the previous section, were measured using a light microscope equipped with a scale, and the average size of the two strains was compared.

### 2.5 Cryopreservation of the PRU strain

The tachyzoites obtained from the previous stage were poured into 1.8‐ml plastic cryogenic vials. FCS (92%) and dimethyl sulfoxide (DMSO, 8%) were added to each vial and the contents were thoroughly mixed on ice. The vials were then stored at −20℃ in an ordinary refrigerator, then at −80℃, and finally they were transferred to liquid nitrogen.

### 2.6 Viability evaluation

After 1 month, the cryopreserved tubes were removed from the liquid nitrogen. The cryopreserved tachyzoites were thawed and washed with RPMI 1640 at 1200 g for 5 minutes (to remove DMSO). The viability and morphology of the recovered tachyzoites were evaluated using a light microscope. Trypan Blue exclusion (0.5% Trypan Blue solution mixed with an equal volume of the suspension) was used to test viability. Viability was also evaluated in a Hela cell culture. The tachyzoites were mixed with the growth medium and added to Hela cells as described above (Mass cultivation). The infectivity potency of the cryopreserved tachyzoites in mice was also examined by injection of 10^4^ tachyzoites intraperitoneally and examining the brains of mice after two months.

## 3 RESULTS

In immunosuppressed mice infected intraperitoneally with tissue cysts, tachyzoites were observed in the peritoneal fluid of 10‐15 days after infection. Tachyzoites of the PRU strain were smaller in size than those of the RH strain (the mean size of PRU and RH strain were 5.5 × 1.5 μm and 6.7 × 2.3 μm, respectively, *P* value = .1). Moreover, mass cell cultivation of the tachyzoites from immunocompromised mice released a lot of viable tachyzoites after 4‐5 days, when all of the cells were ruptured.

**FIGURE 1:**
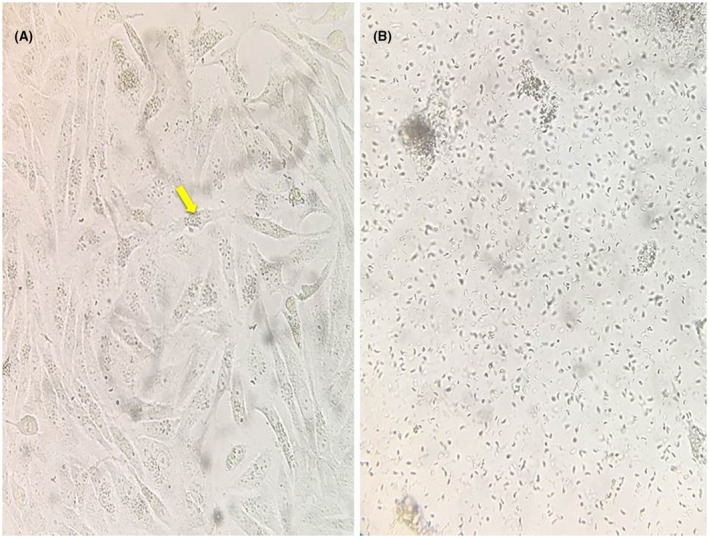
Penetration of HeLa cells by the cryopreserved tachyzoites (A) and release of a lot of viable tachyzoites after rupture of the HeLa cells (B), visualized with a 40× inverted microscope

**FIGURE 2:**
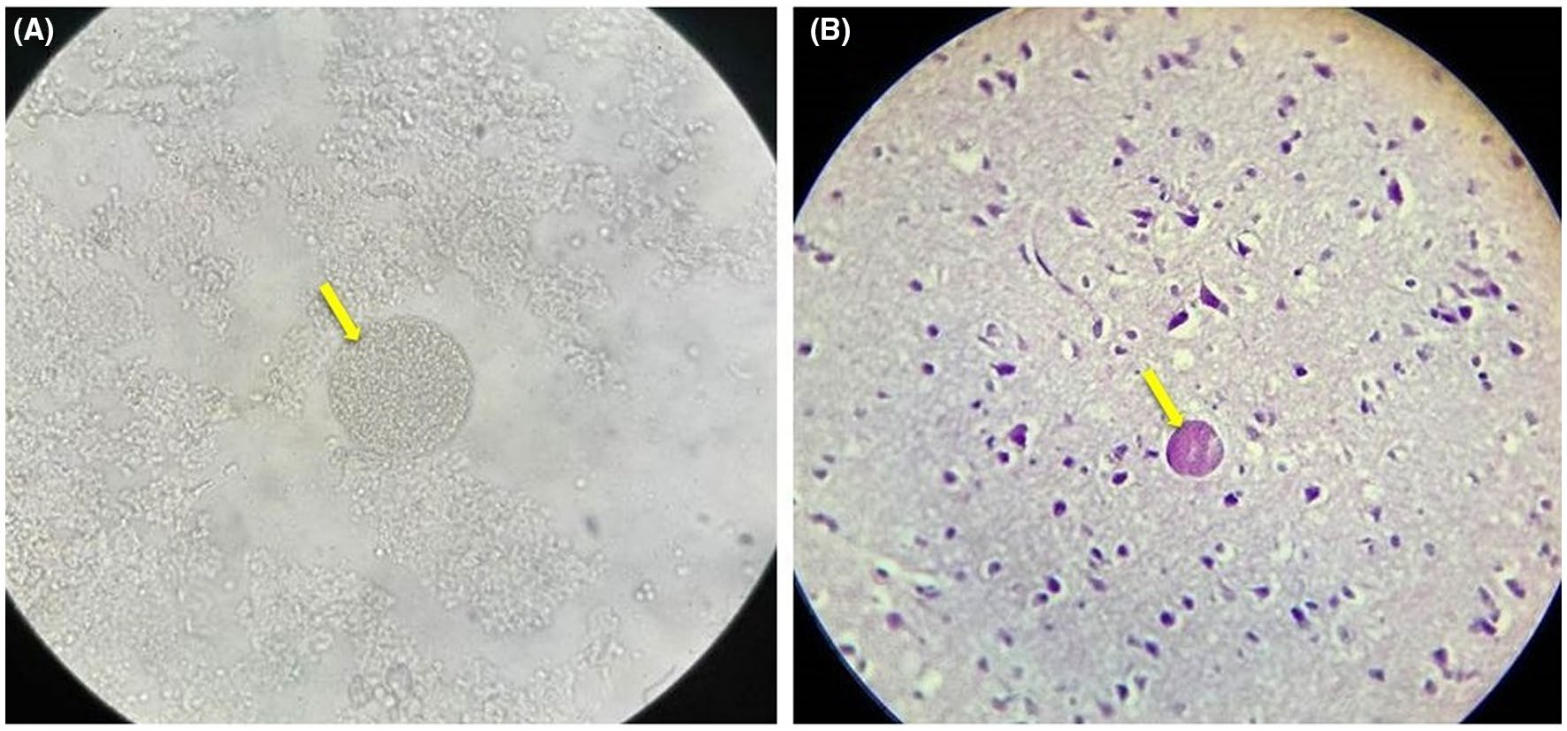
*Toxoplasma* cysts in brains of mice after infection with the cryopreserved tachyzoites. A, Homogenate brain at 100× magnification; B, H&E staining at 40× magnification

In addition, the viability of the cryopreservation method and the viability of the tachyzoites that were maintained in liquid nitrogen were confirmed using Trypan Blue staining and HeLa cell culture. Trypan Blue staining showed 80% viability after cryopreservation. When cell cultures were infected with the cryopreserved tachyzoites, the tachyzoites entered the HeLa cells over 2 days and a lot of viable tachyzoites were released when the all the HeLa cells ruptured after a 7‐day incubation (Figure 1). The infectivity potency of the cryopreserved tachyzoites in mice was also confirmed by observation of cysts in the brain after 2 months (Figure 2).

## 4 DISCUSSION

The present study aimed to describe methods for production and maintenance of *Toxoplasma gondii* tachyzoites of the PRU strain in an animal model and cell culture system. Initially, the immune system of the mice was suppressed using immunosuppressive drugs and cysts of the *Toxoplasma* PRU strain were injected. The infection remained acute in the mice due to their weakened immune system. Ten to fifteen days later, numerous tachyzoites were removed from the peritoneal fluid. In addition, after mass cultivation and cryopreservation, the recovered tachyzoites showed more than 80% viability.

Production and maintenance of *T. gondii* tachyzoites are important for specific antigen preparation, immunization, and therapeutic, biochemical, genetic, and molecular research. The PRU genotype II strain of *Toxoplasma* is very suitable for research on latent toxoplasmosis because this strain immediately progresses to the latent phase due to conversion into bradyzoites in tissue cysts. However, it is difficult to obtain tachyzoites from the PRU strain and therefore most studies have been designed for RH strain maintenance and very few studies have been conducted on the PRU strain.^17^, ^18^

In previous studies, different methods, including trypsin digestion, purification using a 3‐lm filter membrane, CF‐11 cellulose purification, and Percoll solution purification, were used to separate the tachyzoites of *Toxoplasma* from in vivo and in vitro culture systems.^11^, ^16^, ^19^ The trypsin digestion method showed a high salvage rate for tachyzoites purification, but the viability of the purified tachyzoites was low. This method is suitable for antigen analysis.^20^ The 3‐lm filter is the most common purification method for separating tachyzoites. In this low‐cost method, all leukocytes or HeLa cells were removed quickly. However, the tachyzoites recovery rate was low. This method is suitable for purification of small amounts of samples for molecular research.^21^ The CF‐11 cellulose method can also be used for purification of tachyzoites. It removes leukocytes or HeLa cells and is suitable for polymerase chain reaction (PCR) amplification. One of the advantages of this method over the 3‐lm filter is that it harvests a considerable number of tachyzoites, but it is a time*‐*consuming process.^22^ Percoll solution has been also used for separation of human mononuclear leukocytes and purification of tachyzoites. Different concentrations of Percoll solution have been used and 45% iso‐osmotic Percoll solution has shown the best results. However, the tachyzoites recovery rate was low.^23^ In the current study, purification by washing to eliminate the cells and extra debris yielded PRU tachyzoites with a high viability rate. Moreover, when the parasites were introduced into the HeLa cell culture medium after purification, only live, pure tachyzoites to remained in the medium after the first feeding stage. In contrast, the methods employed in other studies caused damage to tachyzoites or the remaining cells and debris. Production and storage of *Toxoplasma gondii* are essential components of all experimental models, genetic studies, biochemical pathways, and drug studies. In the present study, an animal model and in vitro culture system were developed for the production and maintenance of *Toxoplasma gondii* tachyzoites of the PRU strain, allowing their preservation for later use.

## CONFLICT OF INTEREST

The authors declare that they have no conflicts of interest.

## AUTHOR CONTRIBUTIONS

QA and MSB conceived and designed the experiments. MSB, MN, AMS, MM, ND, and SB performed the experiments. MSB and QA analyzed and interpreted the data. QA contributed reagents, materials, analysis tools or data. QA, MSB and ND wrote the original draft, and reviewed and edited subsequent versions.

## ETHICAL APPROVAL

This study was approved by the Ethics Committee of Shiraz University of Medical Sciences (IR.SUMS.REC.1398.678). The guideline of the Institutional Animal Care and Ethics Committee of Animal Experimentation, Shiraz University of Medical Sciences was used to work on animal models in this study.

## Data Availability

The dataset used and/or analyzed during the current study is available from the corresponding author upon reasonable request.

## References

[1] Weiss LM, Dubey JP. Toxoplasmosis: a history of clinical observations. Int J Parasitol. 2009;39(8):895‐901. 19217908 10.1016/j.ijpara.2009.02.004 PMC2704023

[2] Innes E. A brief history and overview of *Toxoplasma gondii* . Zoonoses Public Health. 2010;57(1):1‐7. 10.1111/j.1863-2378.2009.01276.x 19744303

[3] Chowdhury MN. Toxoplasmosis: a review. J Med. 1986;17(5‐6):373‐396. 3295094

[4] Brindle R, Holliman R, Gilks C, Waiyaki P *Toxoplasma* antibodies in HIV‐positive patients from Nairobi. Trans R Soc Trop Med Hyg. 1991;85(6):750‐751. 1801345 10.1016/0035-9203(91)90443-3

[5] Wallace G, Stanford M. Immunity and *Toxoplasma* retinochoroiditis. Clin Exp Immunol. 2008;153(3):309‐315. 18549442 10.1111/j.1365-2249.2008.03692.x PMC2527354

[6] Sibley LD, Boothroyd JC. Virulent strains of *Toxoplasma gondii* comprise a single clonal lineage. Nature. 1992;359(6390):82. 1355855 10.1038/359082a0

[7] Roos DS, Donald RG, Morrissette NS, Moulton ALC. Molecular tools for genetic dissection of the protozoan parasite *Toxoplasma gondii* . Methods Cell Biol. Elsevier. 1995;45(1):27‐63. 10.1016/s0091-679x(08)61845-2 7707991

[8] Bahreini MS, Zarei F, Dastan N, Sami Jahromi S, Pourzargham P, Asgari Q. The relationship between *Toxoplasma gondii* infection in mothers and neonate’s gender. J Matern Fetal Neonatal Med. 2020;33(24):1‐5. 33207997 10.1080/14767058.2020.1849103

[9] Fichera ME, Bhopale MK, Roos DS. In vitro assays elucidate peculiar kinetics of clindamycin action against *Toxoplasma gondii* . Antimicrob Agents Chemother. 1995;39(7):1530‐1537. 7492099 10.1128/aac.39.7.1530 PMC162776

[10] Ashburn D, Evans R, Chatterton J, Joss A, Ho‐Yen D *Toxoplasma* dye test using cell culture derived tachyzoites. J Clin Pathol. 2000;53(8):630‐633. 11002769 10.1136/jcp.53.8.630 PMC1762917

[11] Chatterton JM, Evans R, Ashburn D, Joss A, Ho‐Yen D. Toxoplasma gondii in vitro culture for experimentation. J Microbiol Methods. 2002;51(3):331‐335. 12223293 10.1016/s0167-7012(02)00101-x

[12] Sibley LD, Ajioka JW. Population structure of *Toxoplasma gondii*: clonal expansion driven by infrequent recombination and selective sweeps. Annu Rev Microbiol. 2008;62:329‐351. 18544039 10.1146/annurev.micro.62.081307.162925

[13] Robben PM, Sibley LD. Food‐and waterborne pathogens: you are (infected by) what you eat!. Microb Infect. 2004;6(4):406‐413. 10.1016/j.micinf.2003.12.016 15101398

[14] Döskaya M, Caner A, Can H, Gulce Iz S, Degirmenci A, Guruz AY. Cryopreservation of *Toxoplasma gondii* tachyzoites and tissue cysts. Türkiye Parazitolojii Dergisi. 2013;37(1):44‐46. 23619046 10.5152/tpd.2013.11

[15] Altman D. Cell Culture Protocols, HeLa and CHO cells Woods Hole Physiology Course, 2006. University of Chicago, Chicago, USA: Marine Biological Laboratory; 2006.

[16] Bajelan S, Bahreini MS, Asgari Q, Mikaeili F. Viability and infectivity of *Toxoplasma gondii* tachyzoites exposed to Butanedione monoxime. J Parasit Dis. 2020;44(4):822‐828. 10.1007/s12639-020-01259-9 PMC7430933 32837055

[17] Dempster RP *Toxoplasma* gondii: purification of zoites from peritoneal exudates by eight methods. Exp Parasitol. 1984;57(2):195‐207. 6714360 10.1016/0014-4894(84)90080-8

[18] Saeij JP, Boyle JP, Boothroyd JC. Differences among the three major strains of *Toxoplasma gondii* and their specific interactions with the infected host. Trends Parasitol. 2005;21(10):476‐481. 16098810 10.1016/j.pt.2005.08.001

[19] Dahl R, Johnson A. Purification of *Toxoplasma gondii* from host cells. J Clin Pathol. 1983;36(5):602. 6841653 10.1136/jcp.36.5.602 PMC498296

[20] Derouin F, Mazeron M, Garin Y. Comparative study of tissue culture and mouse inoculation methods for demonstration of *Toxoplasma gondii* . J Clin Microbiol. 1987;25(9):1597‐1600. 3308946 10.1128/jcm.25.9.1597-1600.1987 PMC269290

[21] Radke JR, Gubbels MJ, Jerome ME, Radke JB, Striepen B, White MW. Identification of a sporozoite‐specific member of the *Toxoplasma* SAG superfamily via genetic complementation. Mol Microbiol. 2004;52(1):93‐105. 15049813 10.1111/j.1365-2958.2003.03967.x

[22] Goldman IF, Qari SH, Skinner J, et al. Use of glass beads and CF 11 cellulose for removal of leukocytes from malaria‐infected human blood in field settings. Mem Inst Oswaldo Cruz. 1992;87(4):583‐587. 1343674 10.1590/s0074-02761992000400019

[23] Lee E‐G, Kim J‐H, Shin Y‐S, et al. Application of proteomics for comparison of proteome of Neospora caninum and *Toxoplasma gondii* tachyzoites. J Chromatogr B. 2005;815(1‐2):305‐314. 10.1016/j.jchromb.2004.08.048 15652819

